# Role of malaria partners in malaria elimination in Armenia

**DOI:** 10.1186/s12936-019-2814-y

**Published:** 2019-05-22

**Authors:** Vladimir A. Davidyants, Anatoly V. Kondrashin, Artavazd V. Vanyan, Lola F. Morozova, Natalia A. Turbabina, Ekaterina V. Stepanova, Maria S. Maksimova, Evgeny N. Morozov

**Affiliations:** 1 National Center for Diseases Control and Prevention, Erevan, Armenia; 2 Martsinovsky Institute of Medical Parasitology, Tropical and Vector Borne Diseases, Sechenov University, Moscow, Russian Federation; 3 Department of Tropical Medicine and Parasitic Diseases, Sechenov University, Moscow, Russian Federation; 4 Department of Tropical, Parasitic Diseases and Disinfectology, Russian Medical Academy of Continuous Professional Education, Moscow, Russian Federation

**Keywords:** Malaria, Control, Elimination, Partnership, Intersectoral cooperation, Armenia

## Abstract

Malaria control and preventive activities in the countries of the World Health Organization Region for Europe (WHO/EUR) were strengthened within the framework of the Regional Roll Back Malaria strategy adopted by the member-states at the beginning of the 2000s. A political document “From control to malaria elimination” known as the “Tashkent Declaration” was unanimously endorsed by the member-states of the WHO/EUR with malaria problems in 2005. Since then, considerable progress has been achieved in the countries of the region, signified by the dramatic reduction of malaria incidence in conjunction with the prevention of re-establishment of infection on the territories where malaria was eliminated earlier. Several countries of the region had been certified by the WHO as free of local malaria transmission as a result of the activities of their National Malaria Elimination Programme, Armenia being one of the first in 2011. One of the main lessons learnt during the implementation of the activities by the National Malaria Elimination Programme in Armenia was that the development of an operational plan for malaria elimination required a comprehensive national effort. Full support, both political and financial, from the highest levels of government to smooth coordination between different government ministries, such as Agriculture, Defense, Finance, Health and Policy and Planning and others, was a prerequisite for operational success. The role and place of various partners in the achievement of malaria elimination in the country is discussed in this review.

**Electronic supplementary material:**

The online version of this article (10.1186/s12936-019-2814-y) contains supplementary material, which is available to authorized users.

## Background

Malaria was eliminated in the Republic of Armenia at the beginning of the 1960s. The country was able to maintain its local malaria-free status until 1994 [1, 2]. Transmission of malaria (exclusively *Plasmodium vivax* cases) was resumed in the wake of the collapse of the former USSR, the consequence of a massive earthquake in 1988, the large-scale malaria outbreaks in bordering countries, massive influx of refugees from malaria endemic areas, and socio-economic and political changes in the region [3, 4]. As one of the consequences of such changes, the breakdown of the Ararat Valley drainage system in the early 1990s resulted in elevated ground water levels and created extensive surface water ideal for the breeding of *Anopheles* sp. mosquitoes. These events coincided with the war with Azerbaijan. As a result, there were an appreciable number of malaria cases imported by Armenian servicemen and refugees. According the UNHCR, the war was responsible for displacement of more than 1 million people on both sides [5].

Until 1993, only sporadic malaria cases were imported from abroad, however, importation increased to 195 cases in 1994, and 502 in 1995. Most of the patients were infected during their temporary stay in territories bordering Azerbaijan and Iran. In 1994, the first indigenous case was registered in Armenia since malaria elimination in 1963. Malaria transmission returned to Armenia in full swing by 1996 (149 indigenous cases out of total of 347 cases reported). In 1997, 567 cases (67.4%) were indigenous. In 1998, a total of 1156 cases (542 being indigenous) were reported. Out of a total of 81 districts in the country, 30 recorded malaria cases. In 1998, 89% of the autochthonous cases were detected in the Masis District of the Ararat valley, an area bordering Turkey.

In order to contain malaria epidemic, the Armenian Ministry of Health (MOH) requested support from the WHO in 1996. WHO/EURO and WHO/HQ responded with tailored emergency support over the period 1997–2000. Further supplies for vector control, laboratory diagnosis and malaria treatment were delivered ahead of the peak of the 1998 transmission season, with the support of the United Armenian Fund of the USA. That same year, the MOH and WHO arranged for an extensive training programme on malaria control for the national staff, in cooperation with two WHO Collaborating Centres: the Instituto Superiore di Sanità, Rome, provided training for entomologists and spray men, and the Martsinovsky Institute of Medical Parasitology and Tropical Medicine, Moscow, provided training on malaria diagnosis.

In January 2000, the WHO/EUR assisted the MOH in planning epidemic control measures, and to re-activate the RBM partnership and coordinate activities with the UNICEF and IFRC, the main malaria control partners in Armenia. It also assisted in a preliminary assessment for the establishment of a geographic information system to enhance malaria surveillance in the country. An appeal to support anti-malarial activities in Armenia was developed jointly by the UNICEF, IFRC and WHO, to a total budget of US$ 216,360.

**Fig. 1:**
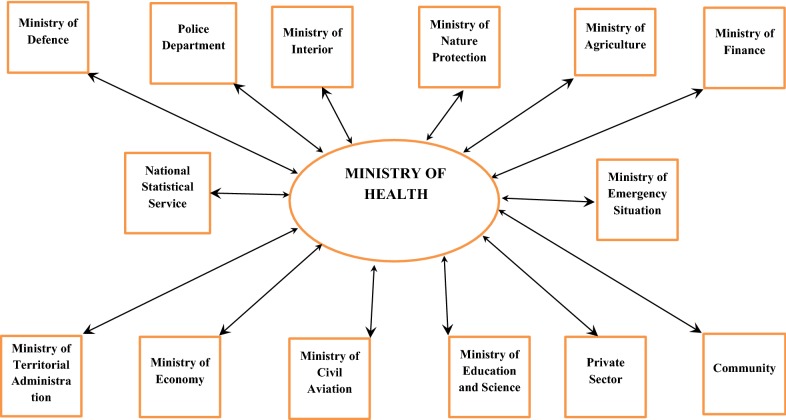
Partners in malaria control/elimination in Armenia

Simultaneously with the efforts to combat the reintroduction of malaria in the late 1990s, the government of Armenia launched an inter-sectoral effort to eliminate malaria in the whole country, the MOH being coordinating the activities of all partners in implementation of all anti malaria activities (Fig. 1). Place and role of Malaria Partnership in malaria control and elimination in Armenia is discussed below.

## Geography of Armenia

**Fig. 2:**
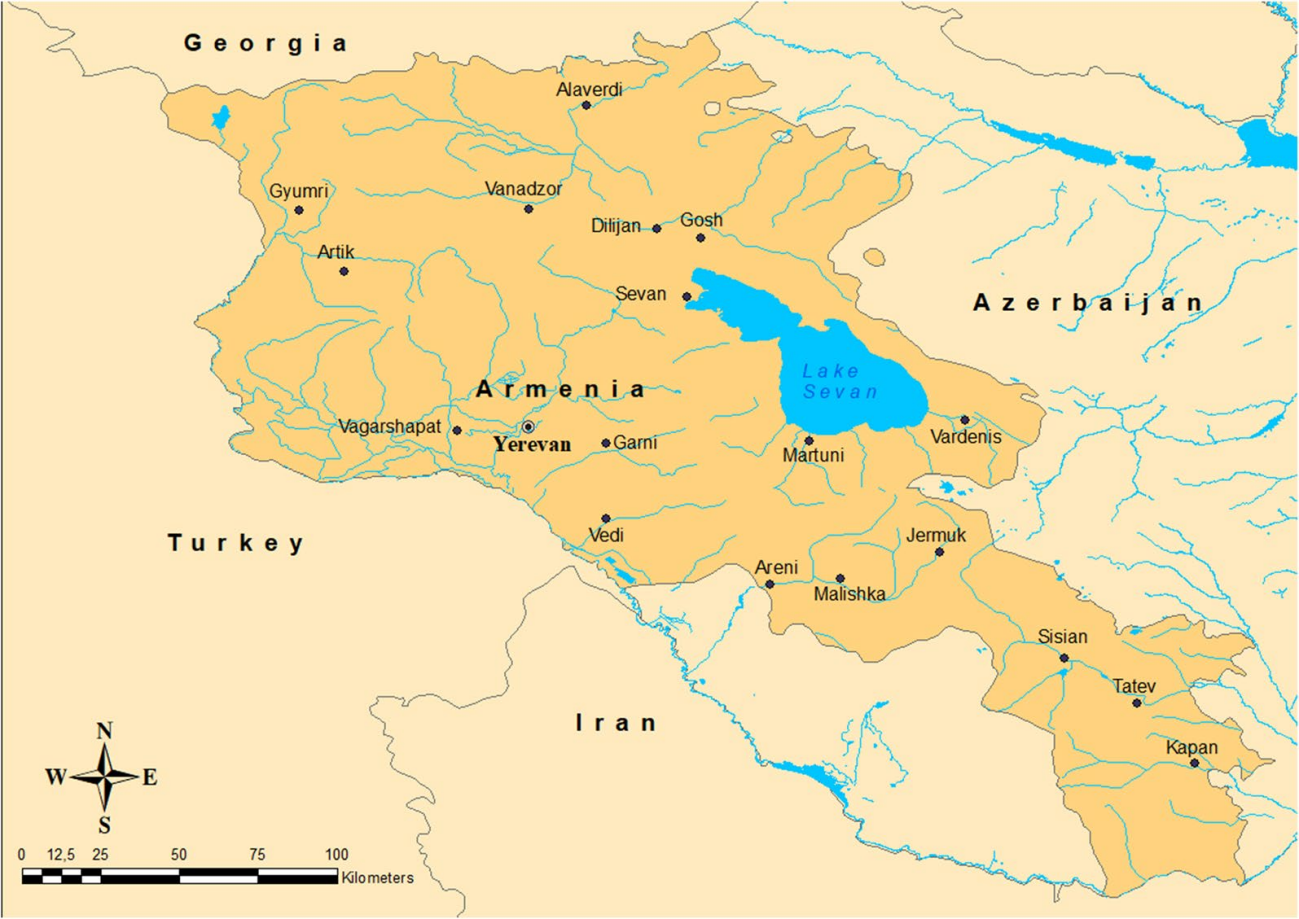
Map of Armenia

Armenia is a landlocked country in Asia, between the Black and Caspian Seas, bordered on the north and east by Georgia and Azerbaijan and on the south and west by Azerbaijan, Iran and Turkey. The terrain is mostly mountainous and occasionally flat, with fast flowing rivers and few forests but with many scattered trees. The climate is highland continental, featuring hot summers and cold winters. The land rises to 4095 m above sea-level at Mount Aragats, and no point is below 400 m above sea level (Fig. 2). Only 16.78% of the land mass is arable with about 2% under permanent cultivation. Irrigated land totals 2860 km^2^.

Armenia is densely populated and highly urbanized. The urban population is 2,081,000 (64.3%) of total country population. Population of the capital city, Yerevan, is 1.1 million. 30% of population lives in formerly malaria-endemic areas, particularly in and around the Ararat Valley. Demographic trends over the last 25 years have been shaped by huge migration of population.

The territory of the Republic is divided into 10 *marzes*, or regions. The territory of each *marz* is further divided into communities.

Like other former Soviet States, Armenia’s economy suffers from the legacy of a centrally planned economy and the breakdown of former Soviet trading patterns. GDP has fallen nearly 60% from its 1989 level. Since 1994, the country has been able to carry out wide-ranging economic reforms which paid off in dramatically lower inflation and steady growth. Armenia has registered strong economic growth since 1995, building on the turnaround that began the previous year, and inflation has been negligible for the past several years. New sectors, such as precious stone processing and jewelry making, information and communication technology, and tourism are beginning to supplement more traditional sectors, such as agriculture in the economy. This steady economic progress has earned Armenia increasing support from international institutions. The IMF, World Bank, EBRD, as well as other international financial institutions and foreign countries are extending considerable grants and loans. Total loans extended to Armenia since 1993 exceed US$ 800 million.

International tourism is a potentially important opportunity for economic development in Armenia. In recent years, the Ministry of Economy has been actively working to enhance the tourism sector. In doing so, there has been close collaboration with malaria elimination efforts, recognizing the potential benefit this could have on the growth of global travel to Armenia. Specific contributions toward elimination have included regulating travel outfitters within the country and overseeing malaria prevention advice to Armenians travelling abroad. Arrival numbers have steadily increased by 20–25% every year since 2001. The number reached 684,000 in 2010 and was expected to climb to more than 800,000 in 2011. The Ministry of Economy has established a goal of three million international visitors by 2030.

## Health system of Armenia

Armenia began reforming its health sector at an early stage following independence. Reform measures included changes to health care delivery in the ambulatory and inpatient settings as well as to the financial and regulatory framework with the overall aim of enhancing efficiency and accessibility of the health care system. The country health reforms are oriented towards international standards.

The health system today comprises a network of independent, self-financing (or mixed financing) health services that provide statutory services and private services. It is divided into three administrative layers: national (republican), regional (marz) and municipal or community. Following the decentralization and reconfiguration of public services after independence, with the exception of the state hygiene and anti-epidemic inspectorate (SHAEI) and several tertiary care hospitals, operation and ownership of health services devolved to local governments (for PHC) and provincial governments (for hospitals).

## Epidemiological features of malaria during resurgence in Armenia

Epidemiology of resurgent malaria in Armenia has been determined by the interaction of factors favorable for malaria transmission as follows:

1. Climatic factors are suitable for seasonal malaria transmission in the plains (particularly in Ararat valley) and elsewhere up to 1200 m above sea level.
2. The presence of one or several efficient malaria vectors, particularly *Anopheles maculipennis*, *Anopheles sacharovi* and *Anopheles superpictus.*
3. Absence of herd immunity to malaria among the population of the country.
4. Availability of numerous mosquito breeding sites due to collapse of irrigation and drainage systems.
5. Massive influx of a source of infection among servicemen, refugees and migrants from neighboring malaria endemic countries.
6. Inadequacies of health services and shortages in supply, equipment, drugs, insecticides.

Among these factors, most important for re-establishment of local malaria transmission had been the massive importation of malaria infection into the country (vulnerability) [6] and collapse of irrigation and drainage systems resulting in creation of numerous malaria vector breeding sites (receptivity) [7].

**Fig. 3:**
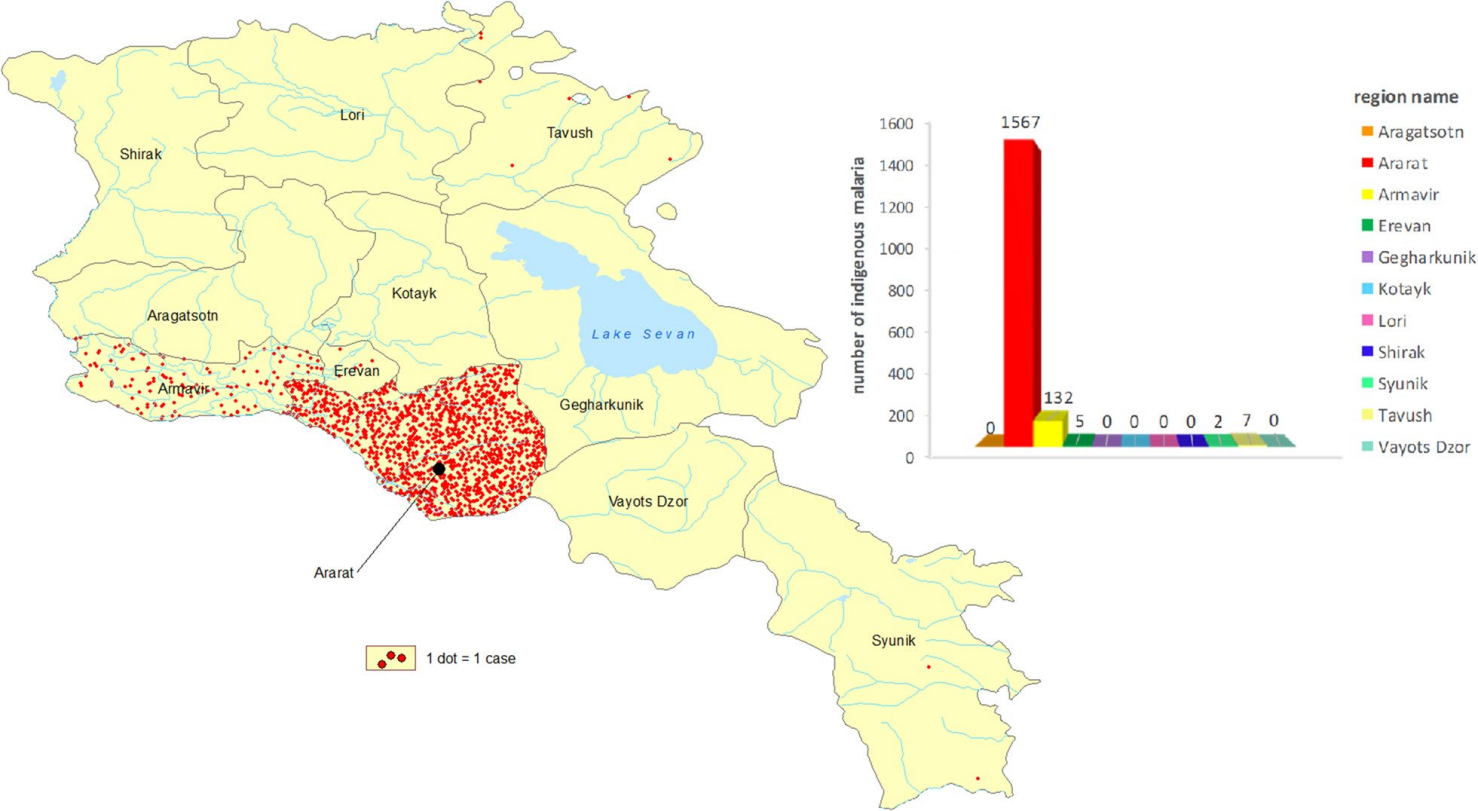
Distribution of indigenous malaria cases in the administrative territories of Armenia

**Fig. 4:**
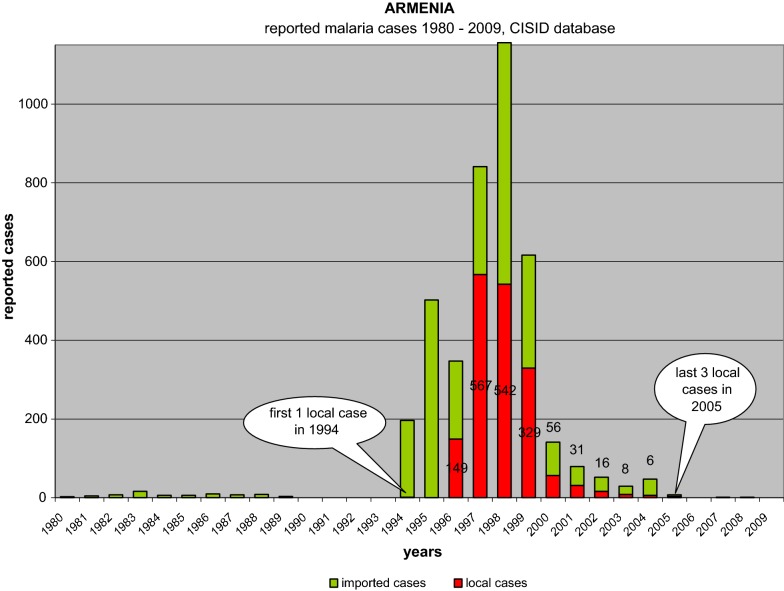
Malaria in Armenia, 1980–2010

Due to impact of anti malaria interventions, greatly supported by external assistance in conjunction with internal resources, the malaria situation started to improve in 1998 when total of 542 indigenous cases of *Plasmodium vivax* were reported in Armenia as compared with a total of 567 cases during the previous year (Fig. 3). The majority of both indigenous and imported cases were found in Masis and Martz Districts in the Ararat valley (Fig. 3). Since 2000, the number of malaria cases (imported and autochthonous) had further demonstrated a declining trend. Only 3 autochthonous cases were reported in 2005—all from a single community. Since then, no autochthonous cases have been reported in the country (Fig. 4) (Additional file 1).

## Role of partners in malaria elimination in Armenia

The Government of Armenia made malaria elimination a priority following malaria burden falls by the year 2005. High-level political involvement proved that it was necessary to secure domestic funding and to ensure flexibility in programme decision-making. It had also demonstrated that the government was willing to do what it takes to ensure elimination and maintain it once it has been achieved. A clear understanding was embedded in the Government that malaria elimination would be unlikely achieved only by the conventional health system: many parts of government and society would have to work together.

The intersectoral work was launched since 1999, by the order of the President of Armenia. Following the order, the Interagency Coordination Committee was established with patronage of Prime Minister who approved several documents regulating intersectoral coordination regarding: (1) the structure of Coordination Committee, (2) distribution of responsibilities, (3) plan of action. The structure of the committee can be seen in Organigramme (Fig. 1). The Cabinet of Ministers Decree regulates distinct distribution of functions between the government bodies, international and non-governmental organizations. Governmental organizations—partners in malaria elimination are headed by the specialists at the rank of the Deputy Minister or by senior managers. The Decree specifies, in full details, the functions of the Ministry of Health, other ministries, municipal bodies of Yerevan city and marzes, other agencies and major concerns.

## Plan of action of the Intersectoral Coordination Committee covers all aspects of intersectoral collaboration

Numerous meetings of the Committee were held to discuss its members’ reports on the work done and other important issues of malaria elimination. Basically, interagency collaboration was set in 2 directions: vertical relationship- local authorities and horizontal relationship—ministries and agencies in the context of relevant objectives. All marzes and Erevan city had their malaria elimination action plan endorsed by respective local authorities and coordinated with local epidemiological surveillance bodies. These plans reflected all principal directions of local authorities aimed at the vector-borne disease control: early detection of cases, laboratory confirmed diagnosis, prompt hospitalization and treatment, environmental management and manipulation, water management, biological control of vectors, sanitation, public awareness and alike.

Once partners were identified, the national malaria programme became the central coordinator to ensure that there was no duplication of work and that the activities of partners were fully aligned with the national strategic plan. In planning multi-year and annual operations, the government established a common work plan with its partners and integrated their activities and milestones into the action plan. The Ministry of Health arranged to meet all partners regularly to ensure that activities are aligned, do not duplicate each other and support the national strategic plan. Twice a year, Ministry of Health conducted together with local authorities monthly malaria protection campaign in accordance with specially developed and approved plans. In 2009 and 2010, round-table discussion, organized by the Ministry of Health with the support from the WHO country office, were held in Yerevan. The round table was attended by all of the country stakeholders: healthcare services, migration service, border service, education, tourism, economic development, mass media. This is another evidence of the importance of intersectoral collaboration and of the adherence of the Ministry of Health to this style of work. Taking into consideration epidemiological situation in the neighbouring countries, border coordination of the malaria-related activities is of particular importance.

Attention attracts a fact that inter-sectoral work had deserved interest of international organizations. In 2002, Armenia was invited to participate at the World Congress of malaria in Washington as an example of comprehensively organized inter-sectoral works against malaria. Vladimir Davidyants, the chief state sanitary doctor of Armenia, Alik Sargsyan, the Governor of the Ararat marz, and Vladimir Tatevosyan, the Head of the reconstruction and collector-drainage networks in the Ararat valley, were invited to the congress.

Numerous meetings of the Interagency Coordination Committee were held to discuss its members’ reports on the work done and other important issues of malaria elimination.

## Role of malaria partners in strengthening malaria surveillance system

Prior to the involvement of malaria partners in malaria control/elimination efforts the health delivery system in the country consisted of the following components:

1. The system of the Ministry of Health;
2. Other state systems performing health services (Defense, Police, Security, Civil Aviation, Education);
3. The system of health facilities established by or functioning under the authority of local municipalities;
4. Private Health facilities;
5. Humanitarian organizations and NGOs performing health projects;
6. Donor-countries and international organizations performing health programmes.

The important role of partners in malaria control was felt particularly in strengthening malaria surveillance country-wide. The backbone of surveillance system is detection of malaria cases by all parts of the health system, e.g. public, private, NGOs, Military, in conjunction with developing reference laboratory capacity for verification of parasitological diagnosis of infection, which is required for decision-making. Therefore compulsory, immediate notification of case(s) is a must [8].

To ensure that malaria is a strictly notifiable disease in the country, the Government issued an order to the effect that all health treatment facilities be it in the governmental or private sector or in specialized services like Defence, Police, National Security, should report on each detected or suspected malaria case to the malaria programme via the MoH. Following the decree by the President, malaria cases could be identified at private sector providers, but should be immediately transferred to publicly-supported infectious disease hospitals. Malaria patients detected at the health treatment facilities in the state systems other than the MoH could be treated there but blood slide had to be sent for confirmation to the Reference Centre. All detected and treated cases in the health treatment facilities in the governmental, non-governmental and private sector must be reported to the MoH within 24 h. The MoH, through its SHAEFI personnel, regularly provides consultative assistance to personnel of private sector engaged in malaria detection activities through field visits, organization of seminars provision of learning aids and alike.

Malaria programme from its side rendered all necessary support to their partners to ensure the quality assurance in the field of malaria diagnosis and treatment of cases. Similarly, the MOH assumed that it would become aware of febrile tourists with suspected malaria through information from hotels, outfitters, and travel agencies. Such a cooperation between various partners in detection and treatment of cases resulted in the improvement of surveillance coverage in time and place. This is exemplified by the fact that the Annual blood examination rate (ABER) was almost doubled in 2007–2010, as compared with previous years (Table 1).

**Table 1:** Annual blood examination rate (ABER %), 2001–2010

Year	Total slides collected	ABER (%)
2001	19,509	3.1
2002	19,707	3.1
2003	20,114	3.7
2004	19,435	3.5
2005	17,720	3.2
2006	19,176	3.6
2007	30,479	5.5
2008	29,095	5.2
2009	30,010	5.3
2010	28,935	5.1

Another consequence of fruitful cooperation of malaria partners was the fact that no single case of asymptomatic carrier of malaria parasite was registered. Absence of asymptomatic cases could be explained by combination of highly efficient laboratory service, equally efficient surveillance system and deployment of Directly-Observed Treatment (DOT) of malaria cases. Molecular diagnostic methods were not used during those times. However, there was an absence of relapses among every treated case during the follow up period of 3 years.

**Fig. 5:**
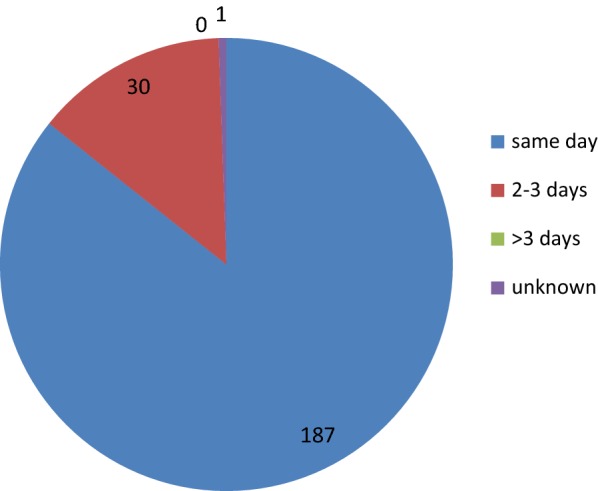
Time lag between confirmation of infection and initiation of malaria treatment

**Fig. 6:**
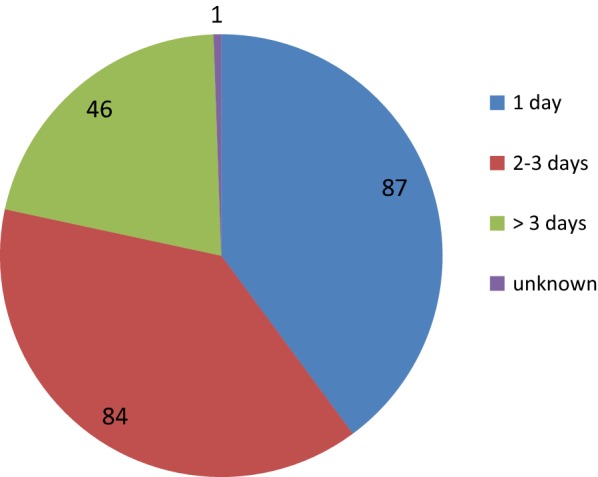
Time lag between reporting of cases and epidemiological investigation in days

Figure 5 depicts the time lag between diagnosis and onset of treatment, which is minimal; Fig. 6 depicts the efficiency of the surveillance and epidemiological investigations of malaria cases and foci.

## Role of malaria partners in prevention local malaria transmission

Soldiers undertaking service in malaria endemic areas abroad, contracting there infection and returning back to their native places were identified as the main source of infection. Dynamics of the spatial distribution of indigenous cases indicate that the first clusters were confined to the areas adjoining Azerbaijan. Commencing from the year 1995, the proliferation of malaria into interior of the country took place on a large scale. By the year 1998, intensive malaria foci were confined to the whole territory of the Ararat valley and beyond.

To prevent malaria contraction among military personnel and border guards, their respective health services carried out malaria prophylaxis during the season of transmission (May–September). Chloroquine single dose (300 mg) once a week was used for the purpose. Decommissioned military personnel who return from endemic areas of neighbouring countries were until 2009 systematically placed on terminal prophylaxis with primaquine (14 days) [9]. Primaquine was used in the standard dose for preventing relapses in *P. vivax* (15 mg base single dose for adults during 14 days). All cases of malaria were treated in the presence of/by medical personnel (DOT). Cases were not examined for G6PD deficiency for want of special tests. No single case of haemolytic anaemia was reported. The list of all decommissioned military personnel with the details of their residential addresses were sent to the MoH for further communication with the local health authorities. A completed primaquine treatment card was needed before the soldiers would receive the military service pass that is required for work clearances or special privileges for entry the university; compliance was, therefore, estimated at 100%. These ex-soldiers were also followed up for 3 years to make sure no late primary attacks or relapses of *P. vivax* occurred. Intensive health education was also provided. The administration of this system was carried out in close cooperation between the Ministry of Defense, the surveillance staff, and the local ambulatory clinic. This terminal prophylaxis system was abandoned in 2010 after malaria was eliminated. The surveillance system is continuing to monitor decommissioned forces for other health concerns, for a period of 3 years upon return. For instance, in 2011 in Shirak, 5400 young men in the 18–20 year age bracket (out of a total province population of 251,540) were called upon for the 2-year obligatory military service through the biannual recruitment drives. Relatively few of the new recruits (perhaps 200/year) would be expected to travel abroad to current or formerly endemic areas. The surveillance system was to keep a close watch on these recruits upon return: actual numbers and names of decommissioned service men had to be provided by the Ministry of Defense directly to the programme so that it could carry out the health investigations and follow up required before the ex-soldiers receive their military service pass.

Another high-risk group for malaria was the border guards, as part of the border area is swampy with extensive potential mosquito breeding sites. There are at present 13 military posts on the border with Turkey, staffed by Armenian and Russian forces. Regularly, about 1500 Armenian jobseekers travel to Georgia and Turkey, countries with residual active malaria transmission. Public health services for the Russian troops are provided by Russia, while the local health services look after the Armenian forces. Unusual health events (including malaria) in the border guards are to be reported by the Russians to the Armenian surveillance system. The border patrols used to take chloroquine as malaria chemoprophylaxis during the transmission season. This practice was stopped in 2007.

Interior Ministry had furnished the MoH with the lists of all such decommissioned persons with the details of their residential addresses. The personnel of malaria control programme provided with primaquine treatment all returnees from military service. As a result of such cooperation, the number of both imported and indigenous cases demonstrated drastic reduction in the country, and the last indigenous case was reported in 2005 (Fig. 1).

## Rehabilitation of drainage system in the Ararat valley

The role of partners such as Agriculture, Territorial Administration, Economy and Finance Ministries in malaria control was particularly important in the rehabilitation of the extensive drainage system in the Ararat Valley (Ararat and Armavir Marzes). Early funding for this restoration was obtained in 1998 from the European Bank for Reconstruction and Development [10] and in 2001 from the World Bank. In consultation with the MoH, priority was given to rehabilitation of the drainage system in the areas most affected by malaria. While in 1998 there were 80,000 hectares of standing water, by 2010 this had been reduced to 15,000 hectares.

**Fig. 7:**
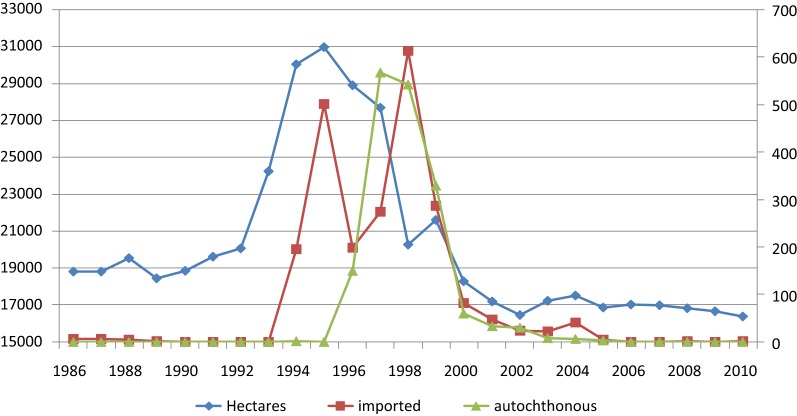
Areas under poor amelioration in the Ararat Valley and total malaria cases, autochthonous and imported, Armenia, 1986–2010

The target for the drainage system was to keep the ground water table at least 2 metres below the surface. Experiences revealed that at levels above 1 m, standing water collections and mosquito breeding become a problem. To check the situation, 450 water table sentinel measurement points were monitored by project staff three times per month, and water table maps were developed based on these observations. In 2010, 72% of the surface area was kept at > 3 m depth, 6% at 2–3 m, 19% at 1–2 m, and 2.85% (total of 394,000 hectares) had water table at less than 1 m depth. These wetter areas included areas surrounding the extensive fish ponds where a lower ground water table was undesirable for economic reasons. There is a strong correlation between the improvement in the drainage system in the Ararat Valley and malaria incidence as can be seen in Fig. 7.

Water management activities were carried out simultaneously with close contact with local administration, department of agriculture, fishery department and health services which had placed malaria control high on the summer monthly agenda from 1998 onwards. Cleaning of drainage canals, land improvement, strengthened surveillance, health education and screening of hospitals and houses near standing water were carried out as intervention measures. Malaria is still on the provincial agenda, even though there have been no cases in recent years. As of 2011, rehabilitation of the drainage system has been completed, and work is continuing with support from various sources, also because of the economic importance of agriculture in Ararat Valley.

The role of other malaria partners in malaria elimination was equally important (Table 2).

**Table 2:** Role of Malaria Partners in Elimination of Malaria in Armenia

Malaria partner	Contribution to malaria elimination
Ministry of Defence	Organization and implementation of malaria epidemiological surveillance in the army; monitoring, analysis, evaluation of detected cases, forecasting and decision-making Entomological vector surveillance of malaria in the army, preparation of passports of water stands located in the territory of military regimens, calculation of malaria seasonal elements, monitoring of Anopheles mosquito population, density, seasonal movement and phenological observations Malaria diagnosis, treatment and follow up of military personnel Seasonal and inter-seasonal chemoprophylaxis Education and awareness of military personnel Monitoring of and ensuring proper implementation of anti-malaria activities in military regiments Implementation of malaria prevention activities in peace-keeping troops
Ministry of Education/Science	Arranged that foreign students from malaria endemic countries were treated at the Medical University policlinic. After a basic medical examination (malaria examination included) they are followed up by the University outpatient clinic and examined for malaria in all cases of fever and upon return after holidays in their native countries. In addition, the Ministry approved malaria-related academic curricula for Yerevan State Medical University and the Institute of Epidemiology, Virology and Medical Parasitology
Interior Ministry and Police Department	There are at present no organized contingents of foreign workers from malaria endemic countries in Armenia. However, there are Armenians who regularly migrate abroad for work(Turkey, Georgia). Migrants need to check in with the family doctor or nurse before departure to get the required travel health certificate and will be checked again on their return by the airport health unit
Ministry of Nature Protection and Ministry of Emergency Situations	Had been actively involved in the process of malariogenic stratification of the territory of Armenia through provision of data on average daily, monthly and annual temperature, as well as on level of precipitation collected at the control sites and reporting these data to the Ministry of Health
Ministry of Territorial Administration	The Personnel of the Ministry were engaged in planning, development and maintenance of drainage systems and land amelioration in the Ararat Valley. It also actively participated through the local governments in implementation of anti-malaria, preventive and anti-epidemiological activities at community (local) level
Ministry of Economy and Ministry of Civil Aviation	Coordinated activities of travel agencies through the Tourism Service of Armenia under Ministry of Economy of Armenia. This cooperation resulted in awareness of travel agencies on malaria risk in endemic areas, and in the awareness of tourists on malaria risk in the destination country. The Ministry of Civil Aviation had ensured proper activity of sanitary and quarantine points (SQP) functioning under the civil aviation system, insect-related examination of international airplanes arriving from endemic areas and monitoring vector-control measures. It also ensured urgent reporting to the MOH (according to the defined mechanism) by airline companies on cases of malaria detected on-board aircraft
Ministry of Agriculture	Apart from active participation in the amelioration of drainage system in Ararat valley, it assumed coordinating role in the judicial use of pesticide for agriculture and public health. It also played a decisive role in the coordination of crop cultivation patterns facilitating the reduction of potential mosquito breeding sites
Ministry of Finance	Ensured state budget allocation for prevention malaria re-establishment within the Health Care System
National Statistical Service	Supported collection and publication on national level the aggregated statistical data on population and communicable diseases, including malaria
Community engagement	It’s role in malaria elimination activities could be seen through the achievement of the malaria programme coverage targets, particularly those related to case detection, DOT treatment (compliance to 17 day treatment at the hospital), and vector control (acceptance of IRS, ITNs, windows screening and distribution of larvivorous fish
Private sector	Participation in detection and diagnosis of suspected malaria cases and in the health education activities

In addition to the contributions of the ministries and government agencies listed here, civil society and professional agencies also took an active role in malaria control. These include the Armenian Medical Association, the Armenian Public Health Association, and the Armenian Public Health Foundation. Such groups contributed to malaria control through organizing continuing education for trained providers; developing and promoting community information, education and communication for malaria prevention and rapid diagnosis and treatment; working with communities of private clinical providers to make sure malaria awareness remains high and reporting complete; and coordinating with travel agencies and outfitters to prepare visitors to meet local and internationally recognized guidelines for preventing travel-associated illness, including malaria.

## Conclusions

Elimination of malaria in Armenia was certified by the WHO in 2011. Since then, the country is in the process of implementation of a comprehensive national plan for prevention of re-establishment of malaria transmission that has the state level approval and strongly builds upon intersectoral collaboration established in the elimination process incorporating a full range of government and non-government entities. As a result of such collaboration, no single case of indigenous transmission of malaria was reported in Armenia until now.

## Additional file

**Additional file 1.** Milestones of National Malaria Programme of the Republic of Armenia.

## Data Availability

The data are available with the National Centre for Disease Control and Prevention of Armenia on reasonable request.

## Abbreviations

UNHCR: United Nations High Commission for Refugees
MoH: Ministry of Health
USA: United States of America
UNICEF: United Nation Children Fund
IFRC: International Federation of the Red Cross and Red Crescent Societies
WHO: World Health Organization
WHO/EUR: European Region of the WHO
SHAEFI: State Hygiene and Anti-epidemic Inspection
RBM: Roll Back Malaria
DOT: Directly-Observed Treatment
IMF: International Monetary Fund
EBRD: European Bank of Reconstruction and Development
ABER: Annual blood examination rate
