# Supplementary material for: Role of malaria partners in malaria elimination in Armenia
Source: Malar J. 2019 May 22;18:178. doi: 10.1186/s12936-019-2814-y (PMC6530163; doi:10.1186/s12936-019-2814-y)
Supplement: Supplementary file 1 — Additional file 1. Milestones of National Malaria Programme of the Republic of Armenia. [file 12936_2019_2814_MOESM1_ESM.docx]

**APPENDIX/ADDITIONAL FILE 1**. Milestones of National Malaria Programme of the Republic of Armenia

End of the 1950s – eradication of malaria on the territory of the erstwhile Soviet Socialist Republic of Armenia

1960-1995 – sporadic imported malaria cases from abroad

1994-1995 – increased importation of malaria cases by the citizens of the Republic of Armenia from abroad

1994- first indigenous *P.vivax* malaria case reported

1996-1998 –large scale *P.vivax* malaria epidemic with overwhelming confinement to Ararat valley

1997-2000 – emergency assistance from the WHO and other international sources to prevent the expansion of malaria to the rest of the country

1998- 2000 – large scale capacity building campaign

2000- development of Plan of Action for malaria control with international assistance

1999-2000 – the Government of the Republic of Armenia launch inter-sector effort to control malaria

1999-2000 – establishment of a National coordination Counsil on Malaria formed by the President and Prime Minister’s decision

May 2001 – the hearing of the malaria problem in the National Assembly of the Republic of Armenia

2002 – establishment of the revised Malaria Control Programme

2003 – approval by the Government the “Malaria Control Targeted Programme 2005-2009” along with national budget allocation

2004 – an inclusion of the National Malaria Control Programme block into the Government Decision No. 100 “Approval of activities aimed at the implementation of the poverty reduction strategic Programme, 2004-2006”

2006 – governmental endorsement of the “Tashkent Declaration from control to malaria elimination”

2006- governmental approval the implementation of the National Malaria Elimination Programme, 2006-2010.

2006 – last indigenous case of malaria in the Republic of Armenia

2011- WHO Certification on indigenous malaria free status on the whole territory of the republic

2011- to date – implementation of the activities of the government-approved Plan of Action on the “Prevention of malaria re-introduction to Armenia.
